# Supplementary material for: Splice-Junction-Based Mapping of Alternative Isoforms in the Human Proteome
Source: Cell Rep. Author manuscript; Available in PMC 2020 Jan 15. (PMC6961840; doi:10.1016/j.celrep.2019.11.026)
Supplement: 2 [file NIHMS1546469-supplement-2.zip › DF1/PXD010154/Testis/Testis_46_PRPF40B_SLAPSTPQAPGIPPPFPPMGLPPMSQRPPAIPPMPPG.pdf]

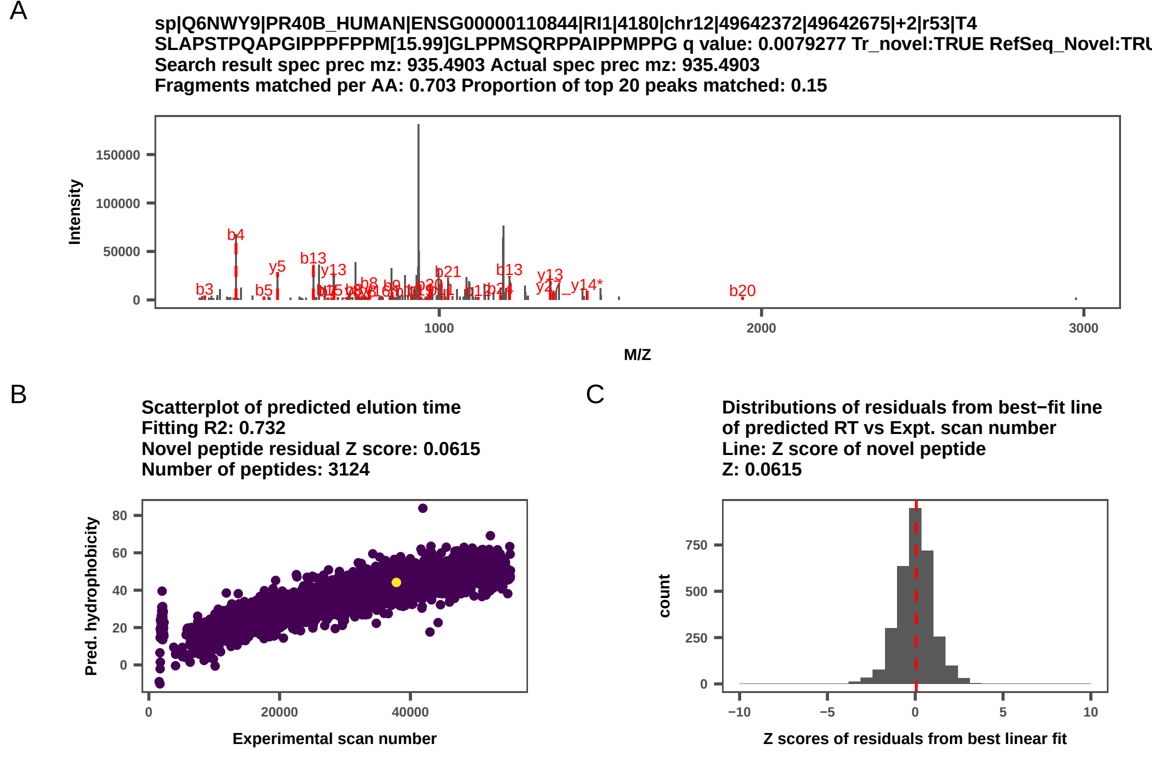
A
sp|Q6NWY9|PR40B_HUMAN|ENSG00000110844|RI1|4180|chr12|49642372|49642675|+2|r53|T4
SLAPSTPQAPGIPPPFPPM[15.99]GLPPMSQRPPAIPPMPPG q value: 0.0079277 Tr_novel:TRUE RefSeq_Novel:TRU
Search result spec prec mz: 935.4903 Actual spec prec mz: 935.4903
Fragments matched per AA: 0.703 Proportion of top 20 peaks matched: 0.15
Intensity
150000
100000
50000
0
b4
y5
b13
y13
b21
b13
y13
y14*
b3
b5
b20
1000
2000
3000
M/Z
B
Scatterplot of predicted elution time
Fitting R2: 0.732
Novel peptide residual Z score: 0.0615
Number of peptides: 3124
Pred. hydrophobicity
80
60
40
20
0
0
20000
40000
Experimental scan number
C
Distributions of residuals from best−fit line
of predicted RT vs Expt. scan number
Line: Z score of novel peptide
Z: 0.0615
count
750
500
250
0
−10
−5
0
5
10
Z scores of residuals from best linear fit
